# Revealing novelty from the southwestern Atlantic, *Yemanjia* gen. nov. and *Olokunococcus* gen. nov. from the coral cyanobiome of the Abrolhos Bank

**DOI:** 10.1111/jpy.70159

**Published:** 2026-04-23

**Authors:** Yuri Ricardo Andrade Aiube, Ana Paula B. Moreira, Taiara Aguiar Caires, Márcio M. B. Tenório, Rodrigo Leão de Moura, Paulo Sergio Salomon

**Affiliations:** ^1^ Genetics Post Graduation Program, Biology Institute Federal University of Rio de Janeiro Rio de Janeiro Brazil; ^2^ Biology Institute, Marine Biology Department and SAGE/COPPE Federal University of Rio de Janeiro Rio de Janeiro Brazil; ^3^ Department of Environmental Engineering Federal University of Bahia Salvador BA Brazil

**Keywords:** 16S rRNA, Aegeococcaceae, cyanobacteria, cyanobiont, Cymatolegaceae, *Favia gravida*, ITS, *Montastraea cavernosa*, *rbc*L, *rpo*C1

## Abstract

Cyanobacteria comprise over 6000 species and inhabit diverse environments, including marine invertebrates such as sponges and corals. High‐throughput sequencing has indicated an abundance of Cyanobacteria communities in these hosts, yet taxonomic resolution has remained low below the phylum level. Most cultured Cyanobacteria from corals have been isolated from black band disease lesions*.* However, many other associated taxa remain unidentified, such as the Cyanobacteria detected with microscopy and isotopic studies near coral symbiosomes. Recently, a polyphasic approach revealed six new genera from sponges. Following a similar strategy—integrating molecular phylogeny, morphology, ecology, and chemotaxonomy—we describe two novel genera and three new species of Cyanobacteria from reef‐building corals of the Abrolhos Banks (southwestern Atlantic). Two filamentous strains were assigned to the new genus *Yemanjia* (Cymatolegaceae), closely related to the genus *Rhodoploca*. A third coccoid strain was assigned to the new genus *Olokunococcus* (Aegeococcaceae), phylogenetically related to *Aegeococcus*. All isolates presented phycoerythrins. The closest formally described relatives of these new taxa are all sponge‐associated, suggesting an evolutionary and ecological link between host and Cyanobacterial lineage. By providing formal taxonomic anchors for coral‐associated Cyanobacteria, our results expand the current knowledge of the coral cyanobiome and facilitate the interpretation of existing and future coral microbiome datasets.

**Abbreviations**
BBD: black band disease
BI: Bayesian inference
BIC: Bayesian information criterion
BLAST: Basil Local Alignment Search Tool
CCMR: Culture Collection of Microalgae
ESQS: Esquecidos Sul reef
ITS: internal transcribed spacer region
MEGA: Molecular and Evolutionary Genetics Analysis
ML: maximum likelihood
PAB: Parcel dos Abrolhos reef
PE: phycoerythrins
*rbc*L: ribulose‐bisphosphate carboxylase gene
*rpo*C1: DNA‐dependent RNA polymerase gene
SAO: South Atlantic Ocean
SEM: scanning electron microscopy
SWAO: Southwestern Atlantic Ocean
UFRJ: Federal University of Rio de Janeiro

## INTRODUCTION

Reef ecosystems exhibit complex symbiotic networks, where corals engage with zooxanthellae (Muscatine & Porter, 1977), green algae (Aiube et al., 2024; Verbruggen et al., 2025; Verbruggen & Tribollet, 2011), fungi, other protists, and prokaryotes (Rohwer et al., 2002). Similarly, sponges constitute highly diverse holobionts (Blackall et al., 2015). These associations foster carbon fixation, nutrient cycling, and protection against pathogens (Ritchie, 2006; Robbins et al., 2019). Although the taxonomy and roles of prokaryotes in coral symbioses have been increasingly studied over the last 2 decades (Rosenberg et al., 2007; van Oppen & Blackall, 2019), Cyanobacterial partners have remained underexplored.

Cyanobacteria form a wide range of symbiotic associations across taxa, from loose interactions to stable endosymbioses (Llamas et al., 2023; Mutalipassi et al., 2021). Microscopy and isotopic studies (Kvennefors & Roff, 2009; Lesser et al., 2004, 2007) revealed cyanobacteria in close proximity to coral symbiosomes, suggesting they play a role in nitrogen provisioning. However, not all associations are beneficial. Cyanobacteria dominate the polymicrobial assemblages of black band disease (BBD; Cooney et al., 2002; Frias‐Lopez et al., 2004), the earliest and most studied coral disease (Antonius, 1973, 1981). They may also contribute to coral overgrowth (Puyana & Prato, 2013; Ritson‐Williams et al., 2005), recruitment inhibition (Kuffner et al., 2006), microbiome disruption (Morrow et al., 2011), and tissue necrosis (Titlyanov et al., 2007). In the Abrolhos Bank, the largest and most biodiverse reef system in the South Atlantic Ocean (SAO; Leão et al., 2003; Simon et al., 2016), we observed summer blooms (Ribeiro et al., 2017) of benthic cyanobacterial mats (Graham & Wilcox, 2000) that, despite being transient, negatively affected coral growth (Ribeiro et al., 2018) and benthic dynamics over time (Teixeira et al., 2021). These concerns led us to investigate the taxonomic profile of those mats and if they could antagonize other organisms. We observed that they were primarily composed of *Moorena bouillonii* (from Parcel dos Abrolhos reef, PAB; Tronholm 2019) or *Okeania erythroflocculosa* (from Pedra de Leste reef; Engene, Paul, et al., 2013) along with *Adonisia turfae* (name not validly published), *Leptolyngbya* sp., and *Halomicronema* sp. These mats exhibited allelopathic effects on *Symbiodinium* sp. CCMR0100, which we isolated from the endangered and endemic *Mussismilia braziliensis*. Experiments with fish and brine shrimp revealed antiherbivory and toxicity properties (Ribeiro et al., 2022).

Despite their ecological relevance, many Cyanobacteria from corals remain unnamed or misidentified. Challenges in isolating and maintaining cultures and a lack of 16S rRNA gene sequences for many strains have hampered taxonomic resolution (Komárek et al., 2014; Mühlsteinov et al., 2018; Palinska & Surosz, 2014). Morphology‐based identification may be unreliable due to cryptic diversity (Mühlsteinov et al., 2018; Woese, 1987; Woese & Fox, 1977). Consequently, reference databases contain numerous mislabeled or unresolved sequences, particularly for problematic genera such as *Lyngbya* (Strunecký et al., 2023). This mismatch is evident when comparing the abundance detected by culture‐independent methods (Moreira et al., 2015; Villela et al., 2024) to the few Cyanobacterial species formally described from corals—namely *Roseofilum reptotaenium* and *Roseofilum corallitycum* (formerly *Phormidium corallitycum*), both from BBD samples (Casamatta et al., 2012). As demonstrated by Konstantinou et al. (2019, 2021), who described six new genera and 10 new species isolated from sponges, a polyphasic approach integrating cultivation, microscopy, phylogenetics, and chemistry can reveal hidden diversity in neglected habitats and hosts. The mutually beneficial association between sponges and their cyanobionts is ancient, and cyanobacteria dominate the outer, light‐exposed surfaces of the host (Hentschel et al., 2006; Taylor et al., 2007). Given the comparable richness of sponge and coral microbiomes (Blackall et al., 2015) and the observation that the proportion of Cyanobacteria in hard corals might reach a similar range (Moreira et al., 2015) to that reported for sponges (reviewed in Hentschel et al., 2006), it is likely that corals also host distinct yet undescribed cyanobionts.

Due to the presence of harmful cyanobacteria in the Abrolhos mats, we employed a polyphasic approach to classify three Cyanobacterial strains isolated from reef‐building corals in the Bank, where nutrient‐rich and turbid waters have been tailoring adapted communities (Leão et al., 2016). Molecular phylogenies and morphological, ecological, and biochemical data supported the delimitation of taxa under the Monophyletic Species Concept (Johansen & Casamatta, 2005), leading to the description of two new genera and three new species within the families Cymatolegaceae and Aegeococcacea, in accordance with the International Code of Nomenclature of Prokaryotes (ICNP). These results expand current knowledge on the coral cyanobiome.

## MATERIALS AND METHODS

### Sampling, isolation and culturing

Specimens of the hard corals *Montastraea cavernosa* and *Favia gravida* were collected in October 2021 and March 2022 in Esquecidos Sul reefs (ESQS), off the Espírito Santo state coast (18°53.888′ S, 39°33.177′ W), and PAB reefs, off the Bahia state coast (17°59.885′ S, 38°40.280′ W), respectively. These sites are part of the Abrolhos Bank, an enlargement of the Brazilian continental shelf in the Southwestern Atlantic Ocean (SWAO), spanning over 20,904 km^2^ and characterized by rhodolith beds, seagrass meadows, algal substrates, and coral pinnacles known as *chapeirões* (Creed & Amado Filho, 1999; Moura et al., 2013). Coral fragments were collected using SCUBA and a hammer and chisel, rinsed with filtered seawater (0.22‐μm), and stored in plastic containers with filtered seawater for transportation. In the laboratory, coral tissues were scraped, homogenized in filtered seawater, and transferred to 96‐well polycarbonate microtitration plates followed by serial dilutions with seawater‐based f/2 medium (Guillard & Lorenzen, 1972) supplemented with 1 μg · mL^−1^ of germanium dioxide (GeO_2_) to inhibit diatom growth (Lewin, 1966). Plates were incubated at 10 μmol photons · m^−2^ · s^−1^, 24°C, and a photoperiod of 12:12 h light:dark, for up to 8 weeks. Upon weekly microscopic inspections, wells with Cyanobacteria growth were selected, and the organisms were isolated through serial dilutions. After 2 weeks, unialgal cultures were subjected to monoclonal isolation. Two filamentous Cyanobacterial strains (CCMR0256 and CCMR0257) were manually sorted by selecting a single filament fragment under the microscope, while a third coccoid strain (CCMR0258) was isolated through serial dilution. Cells were grown in microtitration plates until enough material was available to be transferred to 50‐mL Erlenmeyer flasks and maintained under the same conditions as mentioned above. Cultures were incorporated into the Culture Collection of Microalgae (CCMR) at the Federal University of Rio de Janeiro (UFRJ), Brazil, maintained at the Marine Phytoplankton Laboratory, with strain codes CCMR0256, CCMR0257, and CCMR0258. Strains were fixed in 2% paraformaldehyde and deposited in the collection of the Rio de Janeiro Botanical Garden Research Institute under labels RB 885350 (CCMR0256), RB 885351 (CCMR0257), and RB 885352 (CCMR0258).

**Sampling permit:** No 65055‐8 from Ministério do Meio Ambiente (MMA), Instituto Chico Mendes de Conservação da Biodiversidade (ICMBio), Sistema de Autorização e informação da Biodiversidade (SISBIO).

### Morphological analyses

Strains were grown in fresh medium and analyzed after 2 weeks using an Olympus BX51 microscope (Shinjuku‐ku, Tokyo, Japan) at 200× magnification under bright field and epifluorescence (BP 460‐490 excitation filter). Images were captured with a SONY ILCE‐7M3 camera. Transmission electron microscopy followed Caires et al. (2018). Cells were fixed overnight in Kanovsky's solution (Karnovsky, 1965; modified, final concentrations: 2% glutaraldehyde, 0.05 M sodium cacodylate buffer at pH 7.2, 0.001 M calcium chloride, and 2% paraformaldehyde). Filaments were washed three times with 0.01 M sodium cacodylate buffer and subsequently post‐fixed in 1% osmium tetroxide prepared in the same buffer for 2 h at room temperature. The specimens were washed again with the same buffer and incubated overnight at 4°C in a 2.5% uranyl acetate solution. Dehydration was carried out through a graded acetone series (30%, 50%, and 70% for 5 min each, followed by 90% and 100% for 20 min each). Dehydrated filaments were embedded in Poly/Bed® 812 resin (Polysciences, Warrington, United States). Ultrathin sections were obtained using an ultramicrotome (Leica®, Mannheim, Germany) and post‐stained with 2.5% uranyl acetate and lead citrate. The samples were examined using a JEOL® 1230 transmission electron microscope (JEOL, Tokyo, Japan). For scanning electron microscopy (SEM), samples were fixed overnight as above rinsed three times with 0.01 M sodium cacodylate buffer and dehydrated through an ethanol series (25%, 50%, 70%, 90%, and 100%, each for 15 min). Critical point drying was then performed using a Leica® CPD030 apparatus (Mannheim, Germany). The dried samples were mounted on stubs, coated with a thin gold layer using a Denton Vacuum Desk IV sputter coater (Moorestown, United States), and examined with a JEOL® 6390LV scanning electron microscope (JEOL Ltd., Tokyo, Japan).

### DNA extraction and sequencing

DNA was extracted following Aiube et al. (2024), modified from Schenk et al. (2023), with proteinase K (20 mg · mL^−1^) to enhance cell lyses. Molecular markers included the 16S rRNA gene, ribulose‐bisphosphate carboxylase (*rbc*L) gene, 16S‐23S internal transcribed spacer (ITS) rRNA region, and the DNA‐dependent RNA polymerase (*rpo*C1) gene. Polymerase chain reactions, product purification, and sequences assembly were performed as in Aiube et al. (2024) unless otherwise stated. Primers and amplification conditions are detailed in Table S1. Green Master Mix with GoTaq G2 DNA polymerase (Promega) was employed, except for with *rpo*C1 gene products, which used Phusion DNA polymerase (ThermoFischer, Wilmington, Delaware, United States). The amplification of the *rbc*L gene of the coccoid strain required magnesium (4 mM). Sequencing was performed on a SeqStudio™ Automated Sequencer (Applied Biosystems) at Plataforma de Sequenciamento de DNA, Biophysics Institute, UFRJ. A chimera check was performed using the function chimera.pintail embedded in mothur (Ashelford et al., 2005; Schloss et al., 2009) and vsearch (Rognes et al., 2016). Some amplifications failed successive trials and conditions: the *rpo*C1 gene of CCMR0257 and CCMR0258 and the *rbc*L gene of CCMR0257.

**GenBank accessions:** 16S (PV642589–PV642591), ITS (PV64352–PV64353), *rbc*L (PV662111–PV662112), *rpo*C1 (PV662113).

### Phylogenetic analyses

Raw sequences were edited to generate consensus sequences, which were compared to the GenBank database to retrieve the closest relatives, as in Moreira et al. (2014). The taxonomy of the sequences was checked using the most specific and curated CyanoSeq database (Lefler et al., 2023) as well as AlgaeBase (Guiry & Guiry, 2025). Multiple sequence alignments were conducted using ClustalW (Thompson et al., 1994) in MEGA 7 (Kumar et al., 2016) and MAFFT v7.526 (Katoh et al., 2019). First, sequences were allocated in the phylogenetic space using the 16S rRNA gene. Independent phylogenetic reconstructions were performed also using the ITS rRNA region and the protein coding genes to confirm species delineation. Trees were built using the maximum likelihood (ML) method and Bayesian inference (BI). Maximum likelihood phylogenies were generated with IQ‐TREE (Nguyen et al., 2015), admitting the DNA model selected by ModelFinder (Kalyaanamoorthy et al., 2017) according to the Bayesian information criterion (BIC). Ultrafast bootstrap (Hoang et al., 2017) and the SH‐like approximate likelihood ratio test (Guindon et al., 2010) were employed to evaluate branch support.

For the 16S rRNA gene tree, the General Time Reversible (GTR) model was selected, with an estimated proportion of DNA sites invariant (I) and mutation rates among sites following a gamma distribution (G): GRT + I + G. In the final dataset, there were 62 sequences with 1516 aligned positions, of which 420 were parsimony informative. An expanded 16S rRNA gene tree was built that included all available curated sequences of the orders Nodosilineales and Oculatellales, which frequently appear intermixed in 16S rRNA gene phylogenies. Leptolyngbyales was represented with more members, and sequences of *Roseofilum* (Desertifilales) were also included because the genus includes the only formally described Cyanobacteria from corals. The best‐fit model was Tamura & Nei 3‐parameter (TPM3) admitting I, plus a FreeRate model using four categories (TPM3 + I + R4). The alignment had 110 sequences with 1269 aligned positions (412 parsimony informative). Regarding the ITS rRNA region, the best model was TPM3, using the observed base frequencies (F), admitting I, and the remaining sites evolving at variable rates across four gamma‐distributed categories (G4): (TPM3 + F + I + G4). The final dataset contained 26 sequences with 2602 aligned positions (405 parsimony informative). The phylogeny with the *rbc*L gene sequences was built with the GTR + I + G model and there were 23 sequences with 775 aligned positions (271 parsimony informative). The phylogeny with the *rpo*C1 gene sequences was built with Transitional Model 3 (TIM3) assuming equal base frequencies (e), with I and G4: (TIM3e + I + G4). There were 15 sequences with 887 aligned positions (302 parsimony informative) in the final dataset. Bayesian analyses were conducted using MrBayes v 3.2.7.a (Ronquist et al., 2012), implementing the substitution models obtained in IQTREE ModelFinder and adapted to MrBayes, which resulted in six substitution rates modeled by G + I (Kalyaanamoorthy et al., 2017). For the ITS rRNA region and the *rpo*C1 gene, five million generations were run, whereas for the 16S rRNA and *rbc*L genes, 20 and 10 million generations were run, respectively. Three heated and one cold parallel chain were run for the ITS rRNA region, *rbc*L gene, and *rpo*C1 gene, whereas eight parallel chains (seven heated and one cold) were used for the 16S rRNA gene, with a temperature value of 0.3. All genes were sampled every 1000 generations. The first 25% of the trees were discarded as burn‐in for the ITS rRNA region,  *rbc*L gene, and *rpo*C1 gene, whereas for the 16S rRNA gene, a 50% value was used. Phylogenetic trees were visualized in FigTree v 1.4.4 (Rambaut, 2009). Percentage identities between the gene sequences of the new taxa and the closest phylogenetic neighbors disclosed by the trees were generated using Biopython version 1.81 (Cock et al., 2009). For this analysis, sequences of unclassified or misidentified strains were not considered.

### Secondary structure of the 16S‐23S ITS rRNA region

ITS rRNA region sequences with both tRNAs (tRNA^Ile^ and tRNA^Ala^) were aligned with those of the phylogenetic relatives and reference sequences (Iteman et al., 2000; Konstantinou et al., 2019, 2021). Conserved domains were identified using ClustalW in MEGA 7 (Kumar et al., 2016), and their respective lengths were annotated. Taxonomically important regions (Box B and D1‐D´ helices) were folded in the Mfold WebServer (Zuker, 2003).

### Phycobiliproteins analysis

Extraction and analyses followed Lantoine and Neveux (1997) and Neveux et al. (2006). Briefly, Cyanobacteria cultures were filtered onto glass fiber filters (GF/F), frozen in liquid nitrogen, transferred to glass tubes containing 6 mL of 0.1 M phosphate buffer (pH 6.5), and manually ground with a glass rod. The samples were kept at 4°C in the dark for 4–12 h, followed by centrifugation at ~1100 × *g* (3500 rpm) for 30 min. The supernatant was collected. Excitation (450–580 nm, emission at 605 nm) and emission (530–730 nm, excitation at 495 nm) fluorescence spectra were recorded at 2 nm intervals using a Varian Cary Eclipse spectrofluorometer with slit widths of 5 and 10 nm, respectively. The supernatant was also analyzed with a FEMTO SIRRUS 80ST spectrophotometer to obtain the absorption spectra. All measurements were blank‐corrected (a fresh filter extracted in phosphate buffer) without additional spectral corrections.

## RESULTS

Three Cyanobacterial strains isolated from reef‐building corals were characterized using molecular phylogeny, morphology, ultrastructure, and pigment composition. Combined with the ecological information, these data supported the erection of two new genera and three new species, members of Nodosilineales (family Cymatolegaceae) and Aegeococcales (family Aegeococcaceae). The taxonomic treatment and descriptions presented below follow the guidelines and recommendations outlined by Dvořák et al. (2025).

### Taxonomic descriptions

Class Cyanophyceae

Order Nodosilineales

Family Cymatolegaceae

#### *Yemanjia* Y.Aiube, A.P.B.Moreira & T.A.Caires gen. nov. (Table 1)

**TABLE 1:** Morphological, ultrastructural, and ecological comparison between the novel strains (CCMR) and their closest phylogenetic neighbors.

	Strain	Cell length (μm)	Cell width (μm)	Constriction at cross walls	Apical cell	No. of thylakoids	Thylakoids arrangement	Colony color	Habitat	Lifestyle/host	Location	References
***Yemanjia* and related strains**	***Yemanjia corallina* CCMR0256**	1.2–1.9	1.0–1.2	+	Rounded	3–4	Parietal	Pink	Marine, benthic	Associated to the coral *Favia gravida*	Abrolhos Bank, PAB, Brazil, SWA	This study
	***Y*. *roseoviolacea* CCMR0257**	2.0–2.4	1.3–1.5	+	Rounded	1–2	Parietal	Pink to purple	Marine, benthic	Associated to the coral *Favia gravida*	Abrolhos Bank, PAB, Brazil, SWA	This study
	***Rhodoploca sivonenia* TAU‐MAC 1815**	1.29 ± 0.17	0.88 ± 0.06	++	Rounded	NA	Peripheral	Pinkish	Marine, rocky sub‐littoral zone	Associated to the sponge *Agelas oroides*	Chalkidiki Peninsula, Aegean Sea	Konstantinou et al. (2021)
	***Leptothoe sithoniana* TAU‐MAC 0915**	1.15 ± 0.17	1.3 ± 0.16	+	Rounded	3–4	Peripheral	Reddish	Marine, rocky sublittoral zone	Associated to the sponge *Petrosia ficiformis*	Chalkidiki Peninsula, Aegean Sea	Konstantinou et al. (2019)
	***Leptothoe spongobia* TAU‐MAC 1015**	1.7 ± 0.22	1.08 ± 0 .12	+++	Rounded	3–4	Peripheral	Pinkish	Marine, rocky sublittoral zone	Associated to the sponge *Dysidea avara*	Chalkidiki Peninsula, Aegean Sea	Konstantinou et al. (2019)
	*Leptothoe spongobia* TAU‐MAC 1115	1.8 ± 0.2	1.11 ± 0.13	+++	Rounded	3–4	Peripheral	Pinkish	Marine, rocky sublittoral zone	Associated to the sponge *Acanthella acuta*	Chalkidiki Peninsula, Aegean Sea	Konstantinou et al. (2019)
	***Leptothoe kymatousa* TAU‐MAC 1215**	1.3 ± 0.16	0.89 ± 0.06	++	Rounded	3–4	Peripheral	Pale pink	Marine, rocky sublittoral zone	Associated to the sponge *Chondrilla nucula*	Chalkidiki Peninsula, Aegean Sea	Konstantinou et al. (2019)
	***Cymatolege spiroidea* TAU‐MAC 1315**	1.1 ± 0.15	1.3 ± 0.17	+	Rounded	NA	Peripheral	Pinkish	Marine, rocky sublittoral zone	Associated to the sponge *Aplysina aerophoba*	Chalkidiki Peninsula, Aegean Sea	Konstantinou et al. (2021)
	***Cymatolege isodiametrica* TAU‐MAC 1715**	1.5 ± 0.24	1.4 ± 0.17	+	Rounded	NA	Peripheral	Pinkish	Marine, rocky sublittoral zone	Associated to the sponge *Hexadella racovitzai*	Chalkidiki Peninsula, Aegean Sea	Konstantinou et al. (2021)
	***Salileptolyngbya diazotrophicum* SCSIO**	1.53–2.37	0.93–1.44	+++	+++	NA	Peripheral	Blue‐green	Marine	Planktonic	Northern South China Sea	Zhou et al. (2018)
	***Marileptolyngbya sina SCSIO***	2.23–3.46	1.04–1.73	+++	Rounded	NA	Peripheral	Blue‐green	Marine	Epiphytic on the seagrass *Thalassia hemperichii*	Xincun Bay in Hainan Province, China	Zhou et al. (2018)
	***Nodosilinea nodulosa* UTEX 2910**	1.2–2.4	1.1–1.5	+++	Dome‐shaped	NA	Peripheral	Blue‐green	Marine	Free‐living	South China Sea	Li and Brand (2007) and Perkerson et al. (2011)
	***Haloleptolyngbya alcalis* KR2005/106**	1.2–1.9	1.2–1.9	+++	Rounded	NA	Peripheral	Pale to bright blue	Saline‐alkaline water	Free‐living	Lake Nakuru, Kenya	Dadheech et al. (2012)
***Olokunococcus* and related strains**	***Olokunococcus oblitus* CCMR0258**	1.3–2.1	1.3–2.1	NA	Rounded	NA	Parietal	Pinkish	Marine, benthic	Associated to the coral *Montastrea cavernosa*	Abrolhos Bank, ESQs, Brazil, SWA	This study
	***Aegeococcus thuretii* TAU‐MAC 2015**	2.57 ± 0.3	1.22 ± 0.07	NA	Cylindrical	NA	Parietal	Pale orange	Marine, rocky sublittoral zone	*Associated to the sponge Stryphnus ponderosus*	Chalkidiki Peninsula, Aegean Sea	Konstantinou et al. (2021)
	***Aegeococcus anagnostidisii* TAU‐MAC 0815**	2.14 ± 0.7	0.7 ± 0.2	NA	Cylindrical	NA	Parietal	Pinkish	Marine, rocky sublittoral zone	*Associated to the sponge Axinella damicornis*	Chalkidiki Peninsula, Aegean Sea	Konstantinou et al. (2021)
	*Aegeococcus anagnostidisii* TAU‐MAC 0715	2.66 ± 0.6	1.3 ± 0.1	NA	Cylindrical	NA	Parietal	Pinkish	Marine, rocky sublittoral zone	*Associated to the sponge Axinella cannabina*	Chalkidiki Peninsula, Aegean Sea	Konstantinou et al. (2021)
	***Synechococcus elongatus***	2–9	1.2–3	NA	Cylindrical	NA	Parietal	Blue‐green	Lakes, water springs	Free‐living	All over the temperate zones	Komárek and Anagnostidis (1999)

*Note*: Type strains in bold [slightly constricted = +; constricted = ++; strongly constricted = +++; ESQs, Esquecidos Sul Reefs; NA, not available; PAB, Parcel dos Abrolhos Reefs; SWA, Southwestern Atlantic].

**Figures reference**: 1 and 2

**FIGURE 1:**
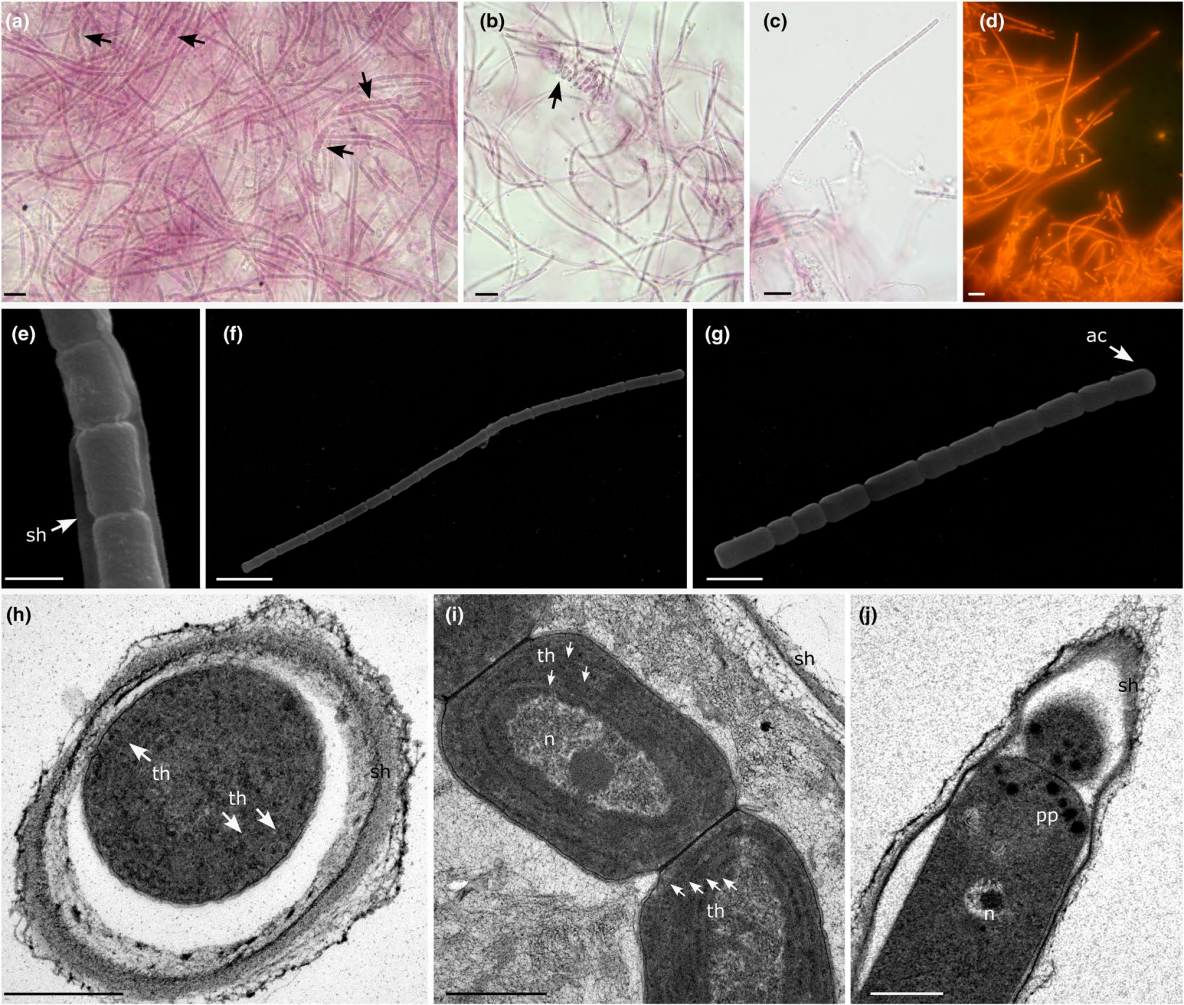
*Yemanjia corallina* (a–c) Light microscopy showing morphology of filaments, arrows indicate coiled ones. (d) Filaments under a fluorescence microscope, BP460‐490 excitation filter. (e–g) Scanning electron microscopy (SEM). (e) Filament, arrow indicates the sheath. (f–g) Trichomes, arrow indicates the apical cell. (h–j) Transmission electron microscopy (TEM). (h) Cross section of a filament. (i–j) Longitudinal sections. Scale bars: A–d = 10 μm, e = 1 μm, f = 5 μm, g = 2 μm, h–i = 500 nm, j = 0.5 μm. N, nucleoid; pp., polyphosphate granules; sh, sheath; th, thylakoids.

**FIGURE 2:**
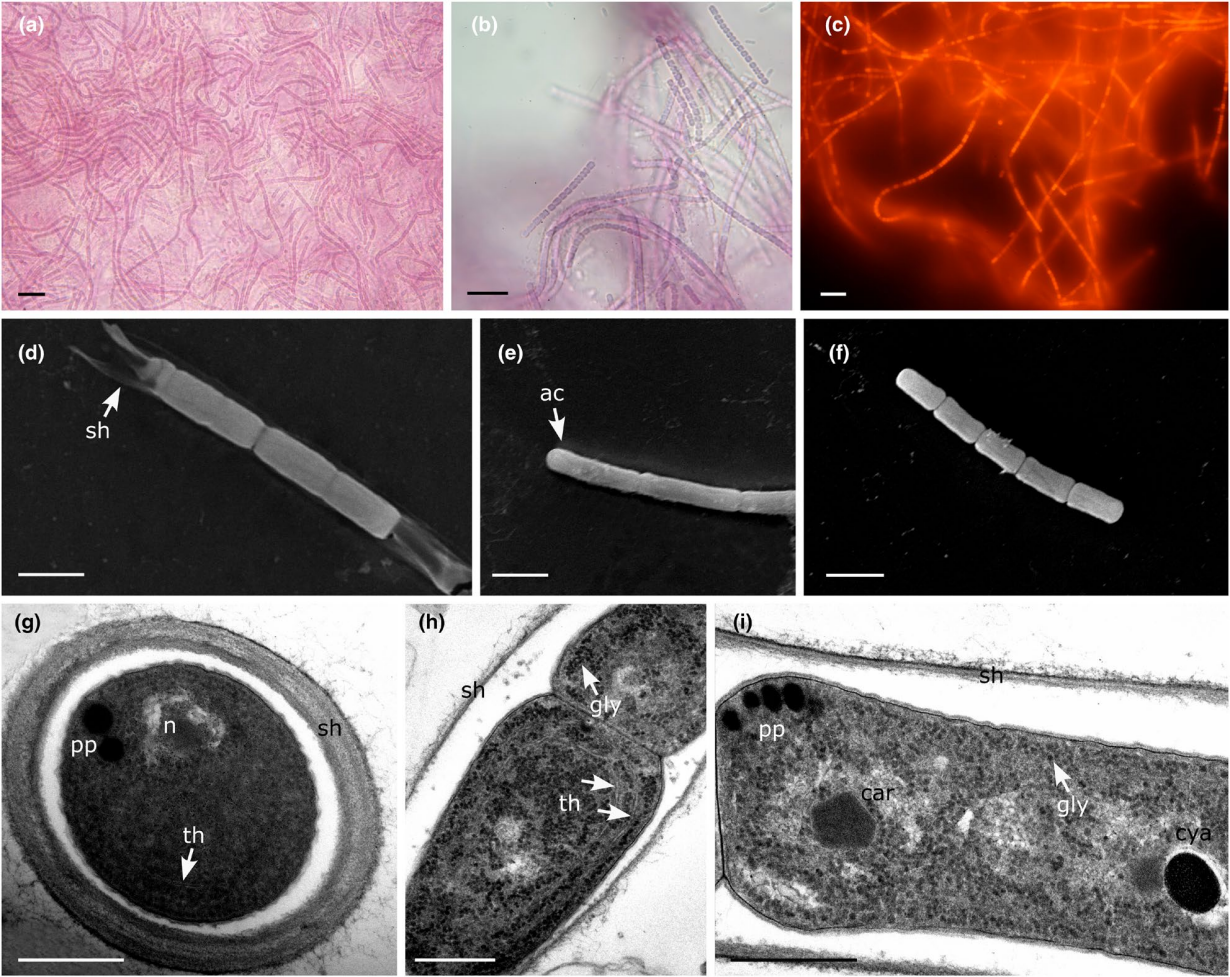
*Yemanjia roseoviolacea* (a and b) Light microscopy showing morphology of filaments. (c) Filaments under a fluorescence microscope, BP460‐490 excitation filter. (d–f) Scanning electron microscopy (SEM). (d) Filament, arrow indicates the sheath. (e) Apical cell. (f) Hormogonia. (g–i) Transmission electron microscopy (TEM). (g) Cross section of a filament. (h and i) Longitudinal sections. Scale bars: A–c = 10 μm, d–f = 2 μm, g–i = 500 nm. Car, carboxysome; cya, cyanophycin granules; gly, glycogen granules; n, nucleoid; pp., polyphosphate granules; sh, sheath; th, thylakoids.

**Description**: Thallus forming pink to purple mats. Sheath thin, firm and hyaline. Straight to wavy or coiled filaments, isopolar, unbranched, 0.8–1.5 μm wide. Trichomes slightly constricted at the cross wall, not attenuated toward ends, 0.7–1.2 μm wide. Cells elongated, 1.0–2.4(−3.8) μm long, up to 2.8× longer than wider. Cell content might be granulated or not, gas vesicles absent. Apical cells rounded to slightly conical‐rounded, without calyptra. Reproduction without the help of necridic cells. Motile hormogonia were observed.

**Diagnosis**: *Yemanjia* differs from *Rhodoploca* in not forming filaments consolidated into partly rope‐like, contorted fascicles, in exhibiting elongated cells, and according to phylogenetic analyses based on the 16S rRNA gene and the 16S–23S ITS rRNA region.

**Etymology**: The word *Yemanjia* refers to Yemanjá, the deity of the ocean worshipped in Brazilian African derived religions such as Umbanda and Candomblé.

**Type species**: *Yemanjia corallina* Y.Aiube, A.P.B.Moreira & T.A.Caires

#### *Yemanjia corallina* Y.Aiube, A.P.B.Moreira & T.A.Caires sp. nov. (Table 1)

**Figure reference:** **1**

**Description:** Thallus forming pink to purple mats. Sheath thin, firm and hyaline. Straight to waved filaments, frequently coiled, isopolar, unbranched, (0.8–) 1.0–1.2 μm wide. Trichomes slightly constricted at the cross walls, not attenuated toward ends, 0.8–1.2 μm wide. Cells elongated, (1.0–)1.2–1.9(−2.4) μm long, 1.5× longer than wide. Cell content homogenous, gas vesicles absent. Apical cells rounded, without calyptra. Reproduction through hormogonia without help of necridic cells.

**Ultrastructural notes:** Three to four parietal thylakoids arranged concentrically, visible in both cross and longitudinal sections in ultrastructural analysis. Only polyphosphate granules were observed near the cross walls.

**Diagnosis:** *Yemanjia corallina* differs from *Y. roseoviolacea* by the presence of frequently coiled filaments, smaller filament diameter (0.8–1.2 μm), cells approximately 1.5× longer than wide, homogeneous cell content, three to four concentrically arranged parietal thylakoids, and according to phylogenetic analyses based on the 16S rRNA gene and the 16S–23S ITS rRNA region.

**Epithet etymology:** The word *corallina* refers to its coral host.

**Holotype:** CCMR 0256; a metabolically inactive mounted specimen derived from strain B6 was deposited in the RB Herbarium—Instituto de Pesquisas Jardim Botânico do Rio de Janeiro, label RB 885350 (!). Brazil, Bahia State, Abrolhos Bank, Parcel dos Abrolhos reefs (17°59.885' S, 38°40.280' W), collected in March 2022, at a depth of ~5 m.

**Reference strain:** CCMR 0256^T^, Culture Collection of Microalgae at UFRJ (CCMR)

Habitat: Marine, benthic, epilithic on the coral host, *Favia gravida*

**Reference sequences:** 16S rRNA gene PV642591, 16S–23S ITS rRNA region PV648354, *rpo*C1 gene PV662113, *rbc*L gene PV662112

**Material analyzed:** CCMR 0256 strain collected in March 2022, at a depth of ~5 m in Parcel dos Abrolhos reefs (17°59.885′ S, 38°40.280′ W), Abrolhos Bank, Bahia State, Brazil.

#### *Yemanjia roseoviolacea* Y.Aiube, A.P.B.Moreira & T.A.Caires sp. nov. (Table 1)

**Figure reference:** 2

**Description:** Thallus forming pink to purple mats. Sheath thin, firm, and hyaline. Straight to waved filaments, isopolar, unbranched, 1.3–1.5 μm wide. Trichomes slightly constricted at the cross wall, not attenuated toward ends, 0.7–1.2 μm wide. Cells elongated, (1.7–)2.0–2.4(−3.8) μm long, 2.8× longer than wide. Cell content granulated, gas vesicles absent. Apical cells rounded to slightly conical‐rounded, without calyptra. Reproduction through hormogonia without help of necridic cells.

Ultrastructural notes: One to two parietal thylakoids arranged concentrically, visible in both cross and longitudinal sections in ultrastructural analysis. Polyphosphate and cyanophycin granules were observed. Cell content was heavily granulated with abundant glycogen granules.

**Diagnosis:** *Yemanjia roseoviolacea* differs from *Y. corallina* by the absence of frequently coiled filaments, in having a larger filament diameter (1.3–1.5 μm), cells approximately 2.8× longer than wide, granulated cell content, one to two concentrically arranged parietal thylakoids, and according to phylogenetic analyses based on the 16S rRNA gene and the 16S–23S ITS rRNA region.

**Epithet etymology:** The specific epithet (ro.se.o.vi.o.la'ce.a) derives from the Latin words *roseus* (pink) and *violaceus* (purple), referring to the pigmentation of the species, which displays pink to purple coloration.

**Holotype:** CCMR0257; a metabolically inactive mounted specimen derived from strain C3 was deposited in the RB Herbarium—Instituto de Pesquisas Jardim Botânico do Rio de Janeiro, label RB 885351(!). Brazil, Espírito Santo State, Abrolhos Bank, Esquecidos Sul Reefs (18°53.888' S, 39°33.177' W) collected in October 2021, at a depth of ~10 m.

**Reference strain:** CCMR0257^T^, Culture Collection of Microalgae at UFRJ (CCMR)

**Habitat:** Marine, benthic, epilithic, associated with the coral *Montastraea cavernosa*

**Reference sequences:** 16S rRNA gene PV642590, 16S‐23S ITS rRNA region PV648353

**Material analyzed**: CCMR0257 strain collected in October 2021, at a depth of ~10 m in Esquecidos Sul Reefs (18°53.888′ S, 39°33.177′ W), Abrolhos Bank, Espírito Santo State, Brazil.

Class Cyanophyceae

Order Aegeococcales

Family Aegeococcaceae

#### *Olokunococcus* Y.Aiube, A.P.B.Moreira & T.A.Caires gen. nov. (Table 1)

**Figure reference:** 3

**FIGURE 3:**
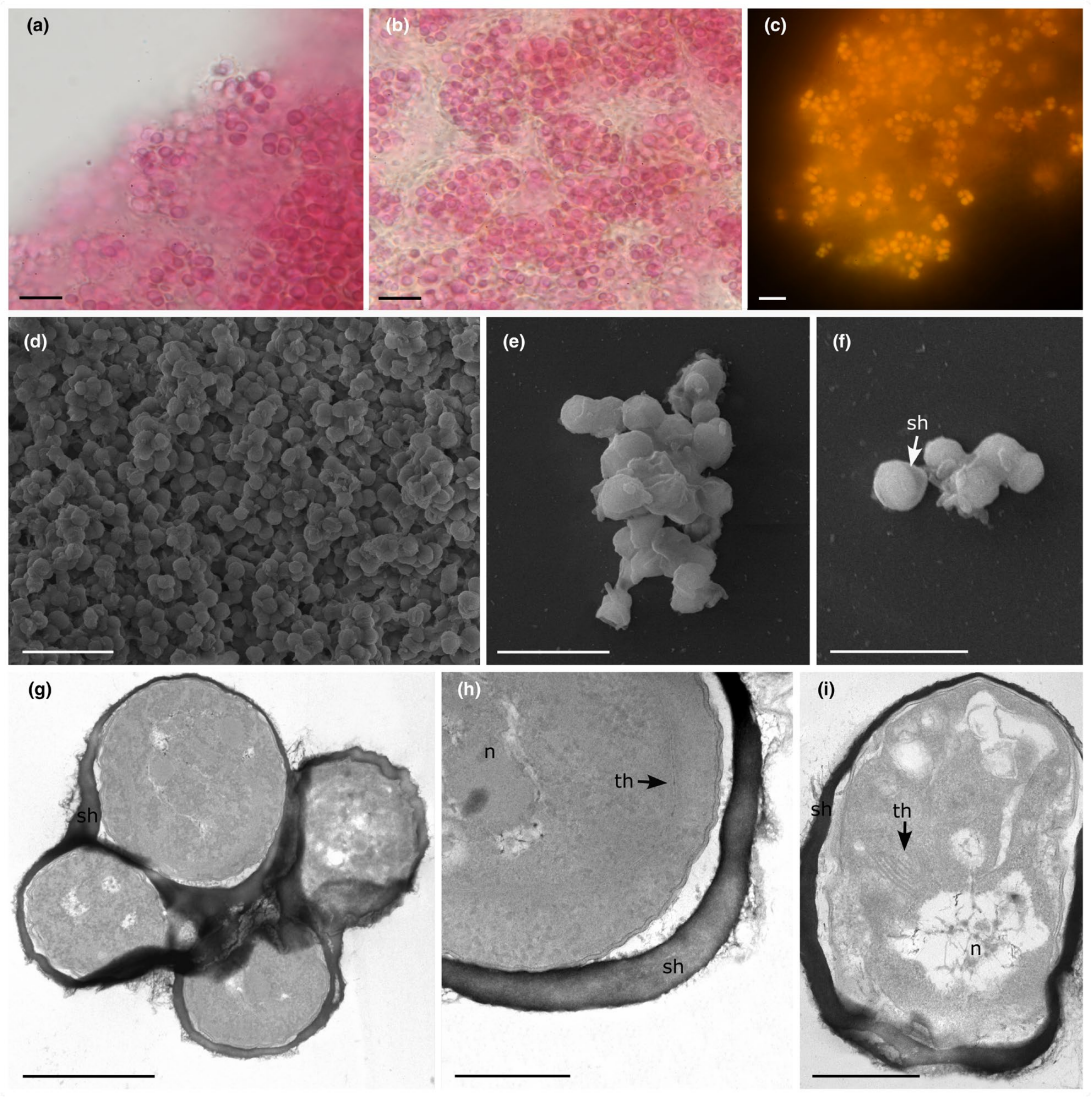
*Olokunococcus oblitus* (a and b) Light microscopy showing morphology of the colonies. (d) Colonies under a fluorescence microscope, BP460‐490 excitation filter. (d and f) Scanning electron microscopy (SEM). (d) General aspect of the colonies. (e and f) Colony details, arrow indicates the sheath. (g–i) Transmission electron microscopy (TEM), cross sections of cells. Scale bars: a–d = 10 μm, e–f = 5 μm, g = 1 μm, h–i = 500 nm. N, nucleoid; sh, sheath; th, thylakoids.

**Description:** Cells irregularly grouped in clusters, surrounded by a mucilage layer. Cells rounded, enclosed by mucilage sheath, 1.3–2.1 μm. Cell content is homogenous and pinkish. Thylakoids predominantly arranged parietally. Reproduction occurs by binary fission.

**Diagnosis:** *Olokunococcus* differs from *Aegeococcus* in having cells always irregularly grouped in clusters, surrounded by a mucilage layer, with rounded cells, and according to phylogenetic analyses based on the 16S rRNA gene and the 16S–23S ITS rRNA region.

**Etymology:** The genus etymology refers to Olokun, the Orisha deity of the bottom of the oceans, worshipped in Candomblé religion in Brazil. The Greek suffix *coccus* (κόκκος) means berry in botanical usage.

**Type species:** *Olokunococcus oblitus* Y.Aiube, A.P.B.Moreira & T.A.Caires

#### *Olokunococcus oblitus* Y.Aiube, A.P.B.Moreira & T.A.Caires sp. nov. (Table 1)

**Figure reference:** **3**

**Description:** Cells irregularly grouped in clusters, surrounded by a mucilage layer. Cells rounded, 1.3–2.1 μm diameter, with distinct individual mucilage sheath. Cells with homogenous content, pinkish. Reproduction by binary fission.

**Ultrastructural notes:** Thylakoid membranes occurred predominantly parietally organized to the cross wall, rarely with irregular arrangement, visible under cross sections in ultrastructural analysis. Polyphosphate and cyanophycin granules were not observed.

**Diagnosis:** *Olokunococcus oblitus* differs from *Aegeococcus anagnostidisii* and *A. thuretii* in having cells irregularly grouped in clusters and surrounded by a mucilage layer, with rounded cells, and in differing from *A. thuretii* by the orange color of the cell content; it further differs from both species according to phylogenetic analyses based on the 16S rRNA gene and the 16S–23S ITS rRNA region.

**Epithet etymology:** *Oblitus* is Latin for “forgotten,” which is the literal translation of the Portuguese name of the reefs (Esquecidos reefs = “Forgotten Reefs”), from where the organism was sampled.

**Holotype:** CCMR0258; a metabolically inactive mounted specimen derived from strain CC was deposited in the RB Herbarium—Instituto de Pesquisas Jardim Botânico do Rio de Janeiro, label RB 885352 (!). Brazil, Espírito Santo State, Abrolhos Bank, Esquecidos Sul Reefs (18°53.888 S, 39°33.177 W) collected in October 2021, at a depth of ~10 m.

**Reference strain:** CCMR0258^T^, Culture Collection of Microalgae at UFRJ (CCMR)

**Habitat:** Marine, benthic, epilithic, associated with *Montastraea cavernosa*.

**Reference sequences:** 16S rRNA gene PV642589, 16S‐23S ITS rRNA region PV648352, *rbc*L gene PV662111

**Material analyzed:** CCMR0258 strain collected in October 2021, at a depth of ~10 m in Esquecidos Sul Reefs (18^°^53.888'S, 39^°^33.177'W), Abrolhos Bank, Bahia State, Brazil.

### Molecular phylogeny and ecology

**FIGURE 4:**
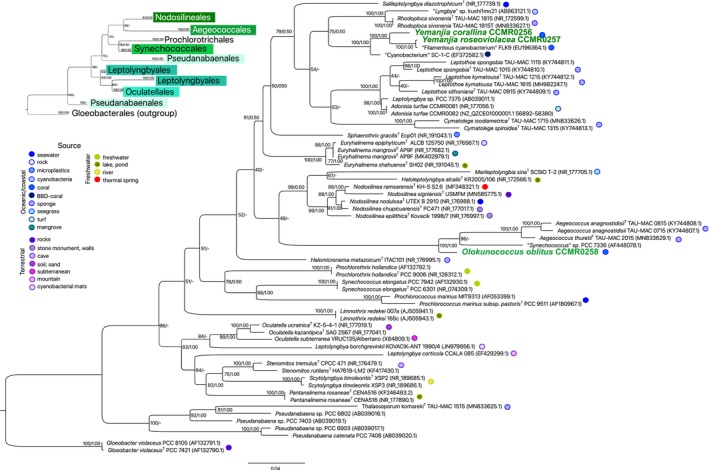
16S rRNA gene phylogenetic tree showing the position of the new taxa (names colored in bold) within the orders Nodosolineales and Aegeococcales. The tree was constructed with maximum likelihood and Bayesian inference methods including sequences belonging to seven orders and *Gloeobacter violaceus* as outgroup. Bootstrap support for ML followed by posterior probability for BI is shown at nodes. Accession numbers are shown in brackets. The names of the type strains are followed by a T (superscript). The names of the unclassified organisms are written in quotes. The smaller tree in the left displays the structure of the tree where the orders are highlighted inside distinct colored boxes. The box colors of Nodosilineales and Aegeococcales correspond to the colors of the names of the new taxa. The source of the strains (when available) is indicated with colored circles, according to the legend.

The phylogenetic reconstruction based on the 16S rRNA gene included the novel strains along with representative taxa of Nodosilineales, Aegeococcales, Prochlorotrichales, Synechococcales, Leptolyngbiales, Pseudanabaenales, and Oculatellales. The filamentous strains isolated from *Favia gravida* (CCMR0256) and *Montastraea cavernosa* (CCMR0257) clustered within the Nodosilineales clade whereas the coccoid strain (CCMR258), isolated from *M. cavernosa*, grouped with Aegeococcales spp. (Figure 4). The new members of Nodosilineales fell within the Cymatolegaceae group with high bootstrap and posterior probability support (100/1.00) representing a new genus named *Yemanjia* with two species: *Y. corallina* (CCMR0256; generitype) and *Y. roseoviolacea* (CCMR0257). The sister branch defined their closest relative as *Rhodoploca sivoneniae* (TAU‐MAC 1815), a monotypic genus isolated from the sponge *Agelas oroides* (Konstantinou et al., 2021). Bifurcating from the *Y. roseoviolacea* (CCMR0257) branch was the sequence (EU196364.1; Myers & Richardson, 2009) of an unclassified strain “Filamentous cyanobacterium FLK9,” retrieved from a cyanobacterial mat on the surface of the coral *Colpophyllia natans*. The cluster formed by *Rhodoploca* and *Yemanjia* included two other sequences of unclassified strains, registered in GenBank as *Lyngbya* sp. kushi1mo21 (AB863121.1) and Cyanobacterium SC‐1‐C (EF372582.1; Myers et al., 2007), which were an isolate from Okinawa reefs and a ribotype from black‐band‐diseased *Siderastrea siderea*, respectively. This bifurcated branch harboring seven sequences (four belonging to the sister genera, *Yemanjia* and *Rhodoploca*, and three sequences from unclassified strains) was reconstructed with identical architecture in the expanded 16S rRNA gene tree (Figure S1). Their parent node encompassed all the other Cymatolegaceae sequences of sponge associated strains of *Leptothoe* and *Cymatolege* (Konstantinou et al., 2019, 2021) as well as *Adonisia* from turf of the Abrolhos Bank (Walter et al., 2020). The strain CCMR0258 was allocated into the Aegeococcales group, hitherto a single‐genus order (Konstantinou et al., 2021), and formed an independent single‐sequence branch, also with maximum statistical support (100/1.00). This node was bifurcated and formed another bifurcated node encompassing both *Aegeococcus* members and another unclassified sequence, “*Synechococcus* sp. PCC 7336” (AF448078.1). Although *Aegeococcus* spp. were also retrieved from sponges (Konstantinou et al., 2021), the strain PCC 7336 was a direct submission, later reclassified, but the name was not validated (Walter et al., 2017). We proposed the novel genus *Olokunococcus*, with *O. oblitus* (CCMR0258) designated as the type species. Phylogenetic analyses placed *O. oblitus* as sister to Aegeococcus *thuretii* (TAU‐MAC 2015), a taxon isolated from the sponge *Stryphnus ponderosus*.

The 16S rRNA gene sequences identities of the three species, compared to the closest tree neighbors, were within or below the range of genus delineation of 94.9% ± 0.4% (Yarza et al., 2008) and below the species threshold of 98.7%–99% (Table 2).

**TABLE 2:** 16S rRNA gene sequences identities (%) between the closest phylogenetic neighbors of *Yemanjia* spp. and *Olokunococcus oblitus* (CCMR).

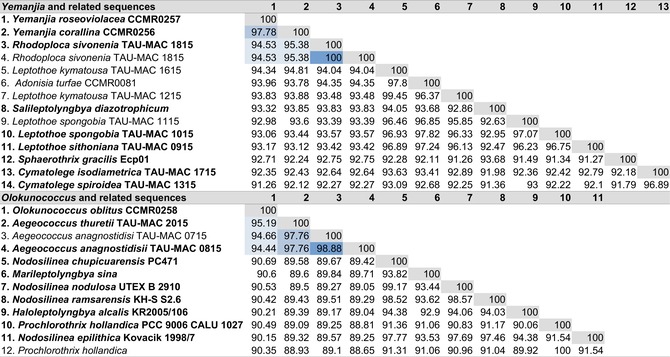

*Note*: Type strains are in bold.

**FIGURE 5:**
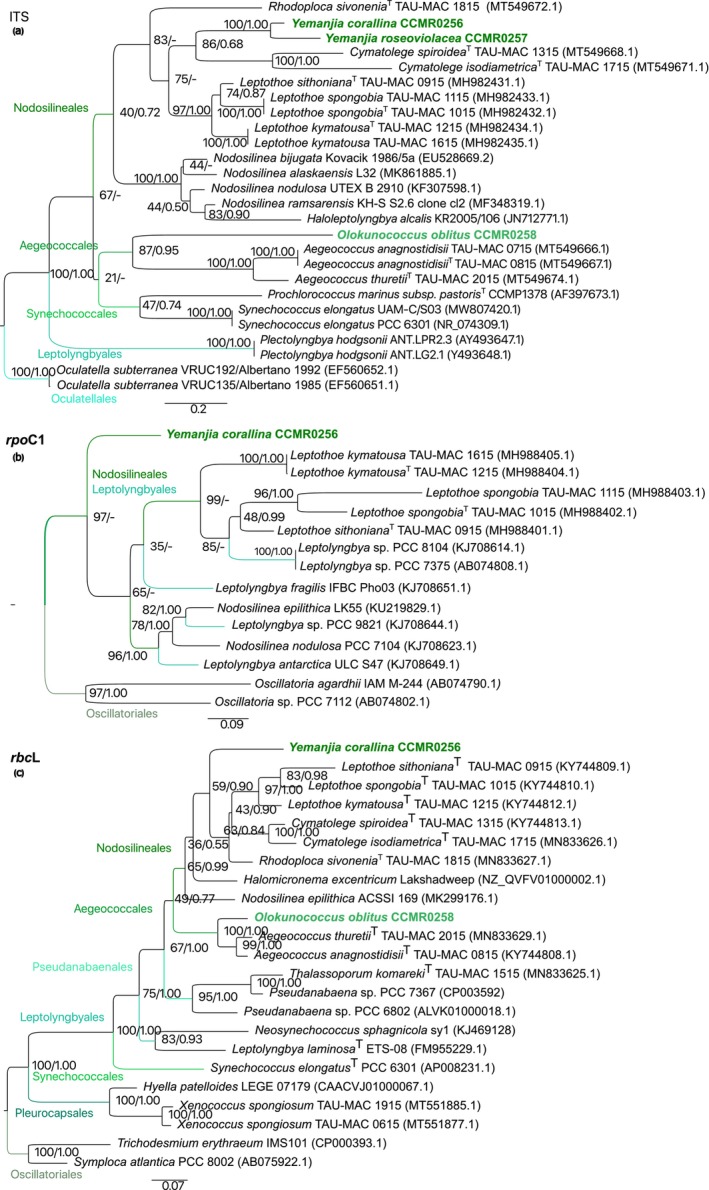
16S‐23S ITS rRNA region and *rpo*C1 and *rbc*L gene phylogenetic trees showing the position of the new taxa (CCMR) within the orders Nodosolineales and Aegeococcales (names colored in bold). (a) 16S–23S ITS rRNA region tree; (b) *rpo*C1 gene tree. (c) *rbc*L gene tree. Trees were constructed with maximum likelihood and Bayesian inference methods. Bootstrap support for ML followed by posterior probability for BI are shown at nodes. Accession numbers are shown in brackets. The names of the type strains are followed by a T (superscript). The colors of the orders' names are the same as the branches where they are placed.

Furthermore, the ITS rRNA region, *rpo*C1 gene and *rbc*L gene phylogenies (Figure 5a–c) supported the robustness and monophyly of the new taxa clusters. In the ITS rRNA region tree (Figure 5a), the *Yemanjia* branch bifurcated from the same node that harbored *Cymatolege* spp., and *Rhodoploca sivoneniae* was an outsider to the Cymatolegaceae branch. The position of *Olokunococcus* in the ITS rRNA region tree mirrored its position in the 16S rRNA gene tree, sharing the node that originated the sister *Aegeococcus* branch (Figure 5a).

The highest sequence identity of both species of *Yemanjia* was to *Leptothoe sithoniana* (TAU‐MAC 0915) followed by the other congeners, *Rhodoploca sivonenia*, and *Cymatolege* spp. (~77%–73% of sequence identity; Table 3). Although all pairs of same species taxa displayed identical sequences (100% of sequence identity), the *Yemanjia* species clustered with total support (100/1.00) and were clearly distinct from each other (85.31% of sequence identity; Table 3). The highest sequence identity of *Olokunococcus oblitus* was with *Aegeococcus thuretii* (TAU‐MAC 2015; 66.86%; Table 3). The identity between the two *Aegeococcus* spp. was ~83%–84% (Table 3).

**TABLE 3:** 16S–23S rRNA ITS region sequence identities (%) between the closest phylogenetic neighbors of *Yemanjia* spp. and *Olokunococcus oblitus* (CCMR).

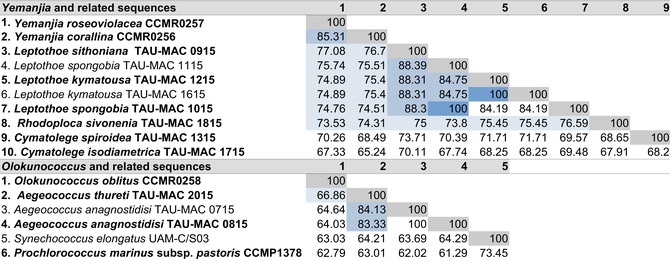

*Note*: Type strains are in bold.

In the *rpo*C1 gene‐based phylogeny (Figure 5b), the *Yemanjia corallina* branch was isolated and outside relative to the node from which all available related sequences arose. The *rbc*L gene tree (Figure 5c) placed *Y. corallina* as outsider to the node that harbored its relatives, members of Cymatolegaceae (*Rhodoploca sivoneniae*, *Leptothoe* spp., and *Cymatolege* spp.). In the Aegeococcales group, the sister genera *Aegeococcus* and the new *Olokunococcus* clustered in distinct branches with total support (100/1.00). Similarity matrices for the new taxa based on the *rpo*C1 and *rbc*L gene sequences are shown in Tables S2 and S3.

### 16S–23S ITS rRNA region secondary structure

**FIGURE 6:**
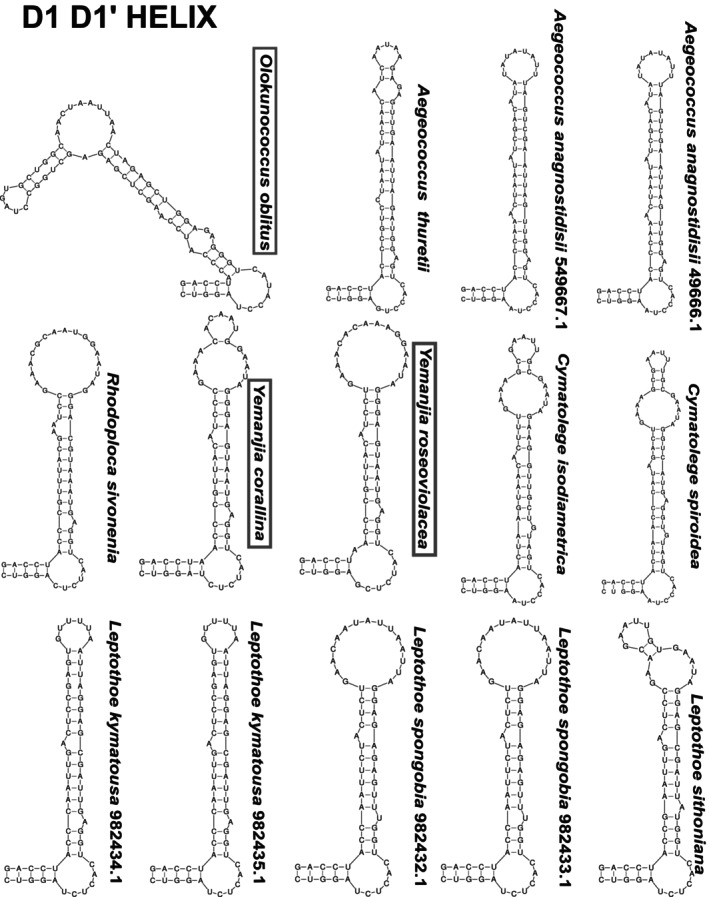
Folded D1–D1′ helix of 16S‐23S rRNA ITS region of the novel taxa (names inside boxes) and their sister species.

**FIGURE 7:**
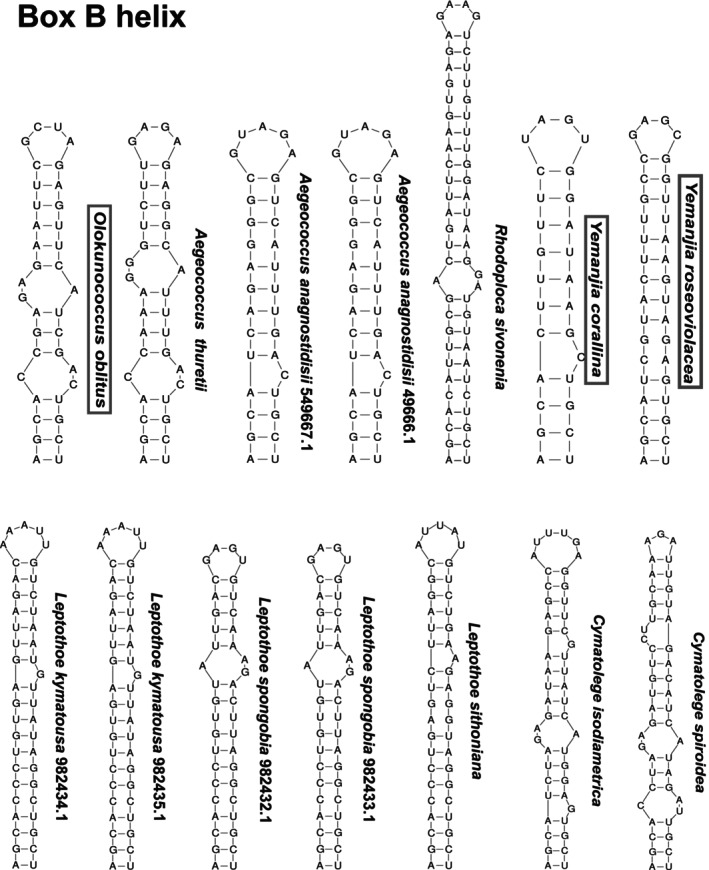
Folded Box B helix of 16S‐23S rRNA ITS region of the novel taxa (names inside boxes) and their sister species.

The ITS rRNA regions were annotated, ranging from 494 to 623 bp in the new strains (Table S4). The D1–D1′ basal clamp (BC) was highly conserved across all ITS rRNA region sequences compared (Figure 6; BC: C = G, C = G, A = U, G = C), reflecting functional constraint and positional homology and supporting orthologous comparison of secondary structures (Villanueva et al., 2024). Regarding the genus *Yemanjia*, the D1–D1′ folding structure had a similar shape compared with those of the other Cymatolegaceae strains, albeit differing from the sister genus *Rhodoploca* when considering the sequences and the number of nucleotides in terminal and internal bulges (Figure 6). When considering their Box B helices, the basal stem was highly conserved among closer taxa, although in *R. sivoneniae*, it was much longer (59) than those from the novel filamentous species (27 and 36 in *Y. corallina* and *Y. roseoviolacea*, respectively; Figure 7). Moreover, both species in the novel genus *Yemanjia* had different overall structures. In D1–D1′ helix, *Y. corallina* and *Y. roseoviolacea* showed contrasting terminal bulge shapes; there were also differences in sequences and in the number of nucleotides of the whole helix (Figure 6). Moreover, the *Y. corallina* and *Y. roseoviolacea* Box B were highly contrasting in length and nucleotide sequence as mentioned above (Figure 7).

*Olokunococcus* exhibited a significantly different D1–D1′ secondary structure compared to those of the other *Aegeococcus* strains, which were much more similar to one another. The differences observed were the number of nucleotides, terminal and internal bulges, unilateral bulges, and internal helix shapes (Figure 7). Another difference between the two genera was observed in the Box B structure: Even though the helix of *O. oblitus* resembled that of *A. thuretii* in shape and number of internal bulges, their overall nucleotide sequences were highly dissimilar (Figure 7).

### Phycobiliproteins

Among phycobiliproteins, phycourobilin exhibits an excitation band around 495 ± 6 nm, whereas longer wavelengths are observed for phycoerythrobilin, that is, between 536 and 565 nm depending on the species. Both excitation and absorption spectra of the three isolates showed single well‐defined peaks of phycoerythrobilin. The *Yemanjia* species showed very similar spectra, with broad peaks and closely spaced maxima at 560 and 562 nm, whereas *Olokunococcus oblitus* displayed a narrower peak, shifted toward 548 nm. No clear signal attributable to phycourobilin was detected, indicating that, if present, concentrations of this chromophore are insufficient to produce a well‐defined peak. (Figure S2).

## DISCUSSION

To date, *Roseofilum* is the only formally described Cyanobacterial genus isolated from corals (Casamatta et al., 2012). The generitype was isolated from *Diploria strigosa* and *Siderastrea siderea* colonies with BBD in a reef site of the Virgin Islands and were shown to induce the disease in mesocosm experiments (Miller & Richardson, 2011; Sato et al., 2009). Prior to this, morphological features and 16S rRNA gene sequence comparisons had attributed BBD to *Oscillatoriales*‐like taxa, particularly *Oscillatoria* sp. Other taxa from BBD assemblages have been tentatively identified (e.g., *Geitlerinema*, *Leptolyngbya*, and *Spirulina*), and their taxonomy remains unresolved. The only exception is *Phormidium* (also reported from BBD), which was reclassified as *Roseofilum* (Casamatta et al., 2012; Meyer et al., 2023; Sato et al., 2016). There is growing evidence that healthy corals harbor Cyanobacteria as well, in the gastroderm (near the symbiosome), potentially fixing nitrogen, but these Cyanobacteria have not been identified (Kvennefors & Roff, 2009; Lesser et al., 2004, 2007). Studies employing high‐throughput sequencing showed that Cyanobacteria figure within the top 10 most abundant phyla in corals (~2%–4% relative abundance; McCauley et al., 2023), but the taxonomic assignments of those Cyanobacteria remain at the genus level at best (Meyer et al., 2023). In addition, many names assigned to sequences in public databases, which are widely used for the taxonomic annotation of culture independent studies, are incorrect or cannot be confirmed because the type material is unavailable or because reliable sequence data are missing. This situation is especially frequent for the ubiquitous morphogenus *Lyngbya* (Strunecký et al., 2023). Furthermore, basic molecular characterization (16S rRNA gene) of most Cyanobacterial generitypes is still lacking (Vondrášková et al., 2025). Metataxonomic assessments generate huge amounts of data (Bednarz et al., 2019; Lema et al., 2012; Moynihan et al., 2022), but they consist of short barcode sequences with reduced phylogenetic discriminatory power and heavily rely on databases that, for Cyanobacteria, remain incomplete and often contain poorly validated taxonomic assignments. Moreover, within a taxonomic level, there can be different levels of sequence identity, depending on the taxa groups (Breitwieser et al., 2019). Given these limitations, the efforts required for culturing and characterizing Cyanobacteria from corals remain essential. Such work is particularly challenging because many coral‐associated strains appear to be host‐dependent or difficult to maintain in culture, even if they are not strict symbionts. Nevertheless, obtaining and characterizing cultured strains is crucial for clarifying the diversity of the coral cyanobiome and for improving our understanding of the ecological roles of coral‐associated taxa, whether harmful, opportunistic, or beneficial. Moreover, sequences derived from well‐characterized isolates and deposited in GenBank improve the accuracy of subsequent taxonomic studies, including those based solely on community DNA sequencing. In addition, Cyanobacteria are a recognized source of compounds of biotechnological interest (Engene, Gunasekera, et al., 2013; Leão et al., 2021), for which the availability of live cultures is essential.

The sister species of the novel strains were all sponge‐associated Cyanobacteria, suggesting some degree of specialization and, probably, the ancestry of the relationships. Other phylogenetically related organisms, thus far unclassified, were retrieved from reef sites and corals, including BBD affected samples (Figure 4). This phylogenetic pattern highlights the link between evolutionary history and ecology and reinforces the urgent need of parallel efforts combining culturing, molecular and genomic characterization of Cyanobacteria. This need is especially pressing in coral holobionts, which have been impacted by climate change and are suffering from a variety of diseases, most of microbial origin (Altizer et al., 2013; Burke et al., 2023; Morais et al., 2022). Within this framework, the SSU rRNA gene phylogeny consistently recovered both *Yemanjia* and *Olokunococcus* as well‐supported monophyletic lineages, fulfilling one of the central criteria for the circumscription of prokaryotic genera and species (Qin et al., 2014; Stackebrandt et al., 2002; Yarza et al., 2008). Regarding the related but unclassified strains, the 16S rRNA gene phylogeny suggests that “Filamentous cyanobacterium FLK9” is probably *Y. roseoviolacea*; “Cyanobacterium SC‐1‐C” likely represents another *Yemanjia* species. Similarly, “*Lyngbya* sp. kushi1mo21” appears to belong to the genus *Rhodoploca*, and “*Synechococcus* sp. PCC 7336” may represent a member of *Aegeococcus* or even a distinct genus. These tentative assignments are based on the 16S rRNA gene phylogeny and sequences divergence; however, the absence of additional phylogenetic markers hinders further taxonomic refinement for these strains. Still regarding the newly described species, their 16S gene sequences were preliminarily screened against an existing coral‐associated metabarcoding library from Abrolhos Bank (Villela et al., 2024). This exploratory mapping revealed the presence of closely related sequence variants in coral samples, underscoring that the newly described taxa likely occur in situ and suggesting that metagenomic approaches could further clarify their spatial distribution and temporal dynamics in coral hosts.

*Yemanjia corallina* and *Y. roseoviolacea* formed an independent cluster shared only with unclassified sequences from corals (strains FLK9 and SC‐1‐C). The expanded 16S rRNA gene tree (Figure S1), with almost all Nodosilineales and Oculatellales members as well as with more *Leptolyngbya* including the *Leptolyngbya* sensu strictu group, clearly supported the proposal of *Yemanjia* as a new genus. In the ITS rRNA region tree, the *Y. corallina* and *Y. roseoviolacea* branch was also independent, but the sister branch was that of the *Cymatolege* spp., not *Rhodoploca sivoneniae*, and the sequence with highest identity (76.70%–77.08%) was that of *L. sithoniana* (type strain) not *R. sivoneniae* (73.53%–74.31%). These identity ranges reinforced the decision to name *Yemanjia* a novel genus (Erwin & Thacker, 2008; Osorio‐Santos et al., 2014). In the *rpo*C1 and *rbc*L gene trees, the *Y. corallina* branch was independent again and very isolated, remaining external to the nodes that originated the other Nodosilineales branches (*rpo*C1 gene) and the other Cymatolegaceae branches (*rbc*L gene). The comparison of the *rbc*L gene sequences showed that the highest identity of *Y. corallina* was with *R. sivoneniae* (86.53%), consistent with the results obtained from the 16S RNA gene sequences comparison. However, unexpectedly, the *R. sivoneniae rbcL* gene sequence showed its highest identity (89.85%) with that of *L. kymatousa* (type strain), rather than with *Y. corallina*, contradicting the relationships inferred from the 16S RNA gene divergence. In summary, the 16S rRNA gene phylogeny indicated *R. sivoneniae* as the closest relative of both *Yemanjia* species, with relatively small genetic distances. However, analyses based on higher resolution markers (ITS rRNA region and *rbc*L gene) revealed a substantially greater divergences between these taxa. Irrespective of the gene, the monophyly of the *Yemanjia* was attained. Altogether the phylogenies and sequences dissimilarities supported the erection of *Yemanjia* as a genus encompassing two species, *Y. corallina* and *Y. roseoviolacea*, which displayed 16S rRNA gene sequences identities (97.78%) far below the species threshold for prokaryotes (98.7%–99%; Kim et al., 2014; Stackebrandt & Ebers, 2006). These filamentous strains could be distinguished from each other and from the related strains also by their ITS rRNA region secondary structure and morphology. The ITS rRNA region structures of both *Yemanjia* strains differed from that of *R. sivoneniae* in the number of nucleotides and sequences in the terminal and internal bulges of the D1–D1′ folding structure. The Box B helix of *R. sivoneniae* was much longer than those of the two *Yemanjia*. Between *Y. corallina* and *Y. roseoviolacea*, there were also differences in length and nucleotides sequence of both helices, reflecting their contrasting structures. Morphologically, *Yemanjia* exhibits some common features of other filamentous Cymatolegaceae, namely thin, firm, and hyaline sheath, apical cells without a calyptra, and a pinkish thallus (Konstantinou et al., 2019, 2021). Nonetheless, *Yemanjia* presented bigger cells, different thylakoid arrangement, and motile hormogonia, a characteristic not yet observed in other filamentous Cymatolegaceae. Thallus color was the synapomorphy of the family until the description of *Vasconcelosia minhoensis*, which presented a *Romeria*‐like morphology, displaying short trichomes, commonly solitary rod or barrel‐shaped cells, and an absence of sheath (de Oliveira et al., 2024; Strunecký et al., 2023). This case illustrated the importance of molecular data to correctly infer phylogenetic relationships in the absence of synapomorphic morphological markers. Compared to *R. sivoneniae*, the generitype *Y*. *corallina* differed by forming straight to coiled filaments and an unstructured thallus, whereas *R. sivoneniae* exhibited straight to curved filaments consolidated into fascicles, often forming rope‐like contortions (Konstantinou et al., 2021). *Yemanjia corallina* was distinguished from *Y. roseoviolacea* in several characteristics, exhibiting frequently coiled filaments, which were not observed in *Y. roseoviolacea*. Additionally, *Y. roseoviolacea* had larger measurements, with a greater filament diameter and elongated cells up to 2.8 times longer than wide, whereas Y. *corallina* cells were only up to 1.5 times longer than wide. Furthermore, *Y*. *corallina* presented more thylakoids, and *Y. roseoviolacea* displayed granulated cell content with abundant glycogen granules, in contrast to *Y*. *corallina*, which exhibited homogeneous cell content.

*Olokunococcus oblitus* was related to the sponge‐associated members of *Aegeococcus* in all phylogenies. The 16S rRNA gene identities to the closest sequences, *Aegeococcus thuretii* followed by *A*. *anagnostidisii* (94.44%–95.19%), fell in the genus threshold range. However, the ITS rRNA region and *rbc*L gene sequence identities of *O*. *oblitus* compared with the *Aegeococcus* spp. were significantly lower than those of the intrageneric identities (between *A. thuretii* and *A*. *anagnostidisii*). For the ITS rRNA region, the intergeneric identity range of 64.06%–66.86% separated the two taxa (Erwin & Thacker, 2008; Osorio‐Santos et al., 2014) whereas the intrageneric identify (between *A. thuretii* and *A*. *anagnostidisii*) was higher (83.33%–84.13%). For the *rbc*L gene, the same comparisons resulted in 90.54%–90.79% versus 95.04%, respectively. In addition, remarkable differences between *O*. *oblitus* and *Aegeococcus* spp. were observed in the ITS rRNA region secondary structures and morphology. The D1–D1′ helix of *Olokunococcus* was longer than the helices of *A. anagnostidisii* and *A. thuretii* and contained a prominent bilateral internal bulge, producing a conspicuous helix‐shaped absent in *Aegeococcus*. The overall nucleotide sequences were also dissimilar. The folded structure of the Box B helix differed between *Olokunococcus* and *Aegeococcus* in both length and nucleotide sequence. A single internal bulge was observed in *A. anagnostidisii*, whereas two were present in *O. oblitus* and *A*. *thuretii*; however, their nucleotide composition and structural context differed substantially. The nucleotide sequences in the terminal bulges also differed between the two genera. Regarding morphology, *O*. *oblitus* formed mucilaginous colonies, not reported for *Aegeococcus*, and the cells were rounded (diameter: 1.3–2.1 μm), whereas the cells of the sister genus were cylindrical, longer (1.17–4.42 μm) than wide (0.61–1.3 μm). In this integrative taxonomic framework, multi‐gene phylogenies constituted the primary evidence for taxon novelty, whereas ITS rRNA region secondary structure, morphological traits, and pigment analyses provided complementary support.

Phycobiliproteins—particularly phycoerythrins (PEs)—are the main accessory pigments of cyanobacteria in tropical and subtropical waters, and their spectral diversity largely reflects the relative abundance of phycourobilin and phycoerythrobilin. Variation in PE excitation spectra has been widely applied as a chemotaxonomic marker, enabling the discrimination of *Synechococcus* lineages and the differentiation of filamentous diazotrophic taxa such as *Trichodesmium* and *Richelia* (Lantoine & Neveux, 1997; Neveux et al., 2006, 2009). Spectral differences in phycobiliproteins have also been shown to mirror evolutionary relationships among Cyanobacteria (Apt et al., 1995), underscoring their value for species‐level resolution within natural Cyanobacterial assemblages.

The shared spectral features among the isolates (*Yemanjia* and *Olokunococcus*) point to a functional convergence driven by the coral microhabitat. Nevertheless, this convergence operates at the level of ecological function, whereas discrete differences in excitation peak shapes and positions reflected structural variations in the PEs of *Yemanjia* and *Olokunococcus*, preserving taxonomically informative traits. Studies focusing on PE fluorescence in coral‐associated Cyanobacteria remain scarce (Mutalipassi et al., 2021). A notable exception is the study by Lesser et al. (2004), who investigated the daytime orange fluorescence of *Montastraea cavernosa*—the coral host of *Olokunococcus*—and demonstrated that this signal was attributable to PE rather than to coral‐derived proteins. They localized PE‐reactive coccoid Cyanobacterial cells within coral host cells, which were also reactive to a nitrogenase antibody, suggesting that this endosymbiotic cyanobiont may contribute fixed nitrogen to the coral and potentially form a stable long‐term association within host tissues. Overall, the spectral characterization of phycobiliproteins, when combined with molecular data, provides valuable insights not only into the taxonomy and photoacclimation strategies of coral‐associated Cyanobacteria but also into their ecological roles within the coral holobiont.

## CONCLUSIONS

Discovery of novel, coral‐associated Cyanobacterial lineages contributes to greater understanding of the coral microbiomes. Our results reinforce the strength of a polyphasic framework for resolving cyanobacterial taxonomy in ecologically complex systems. Beyond their taxonomic significance, these coral‐associated Cyanobacteria represent previously uncharacterized components of reef holobionts, with functional roles, biogeographic patterns, and biotechnological potential that remain largely unexplored. Future genomic and metagenomic investigations will be essential to elucidate how these lineages contribute to coral resilience, nutrient cycling, and reef ecosystem functioning under escalating anthropogenic pressures.

## AUTHOR CONTRIBUTIONS

**Yuri Ricardo Andrade Aiube:** Conceptualization (equal); formal analysis (equal); investigation (equal); writing – original draft (equal); writing – review and editing (equal). **Ana Paula B. Moreira:** Conceptualization (equal); formal analysis (equal); investigation (equal); supervision (lead); writing – original draft (equal); writing – review and editing (equal). **Márcio M. B. Tenório:** Formal analysis (supporting); investigation (supporting); writing – review and editing (supporting). **Taiara Aguiar Caires:** Formal analysis (supporting); investigation (supporting); writing – original draft (supporting); writing – review and editing (supporting). **Rodrigo Leão de Moura:** Funding acquisition (lead); resources (lead); writing – review and editing (equal). **Paulo Sergio Salomon:** Funding acquisition (lead); resources (lead); writing – original draft (equal); writing – review and editing (equal).

## Supporting information


**Figure S1.** Expanded 16S rRNA gene phylogenetic tree showing the position of the new taxa (names colored in bold) within the orders Nodosolineales and Aegeococcales. The tree was constructed with maximum likelihood method including sequences belonging to seven orders and *Gloeobacter violaceus* as outgroup. All available sequences of Nodosolineales and Aegeococcales were included as well as a more representative subset of Leptolyngbyales (relative to Figure 4) and *Roseofilum* sequences. Bootstrap support followed by SH‐like approximate likelihood ratio is shown at nodes. Accession numbers are shown in brackets.

**Figure S2.** Fluorescence‐excitation (a) and absorption (b) spectra of phycoerythrins from *Yemanjia corallina*, *Y. roseoviolacea*, and *Olokunococcus oblitus*. Spectra were normalized at the excitation maximum. A single well‐defined phycoerythrobilin peak was observed in all isolates, with no clear signal attributable to phycourobilin.

**Table S1.** Primers and amplification conditions

**Table S2.** *rpo*C1 region gene sequences identities (%) between *Yemanjia corallina* and its closest phylogenetic neighbors. Type strains are in bold.

**Table S3.** *rbc*L gene sequences identities (%) between the closest phylogenetic neighbors of *Yemanjia corallina* and *Olokunococcus oblitus* (CCMR0258). Type strains are in bold.

**Table S4.** Annotation of the 16S–23S ITS rRNA regions and corresponding lengths.
