# Supplementary figures and images for: Revealing novelty from the southwestern Atlantic, Yemanjia gen. nov. and Olokunococcus gen. nov. from the coral cyanobiome of the Abrolhos Bank
Source: J Phycol. 2026 Apr 23;62(2):533–55. doi: 10.1111/jpy.70159 (PMC13103685; doi:10.1111/jpy.70159)

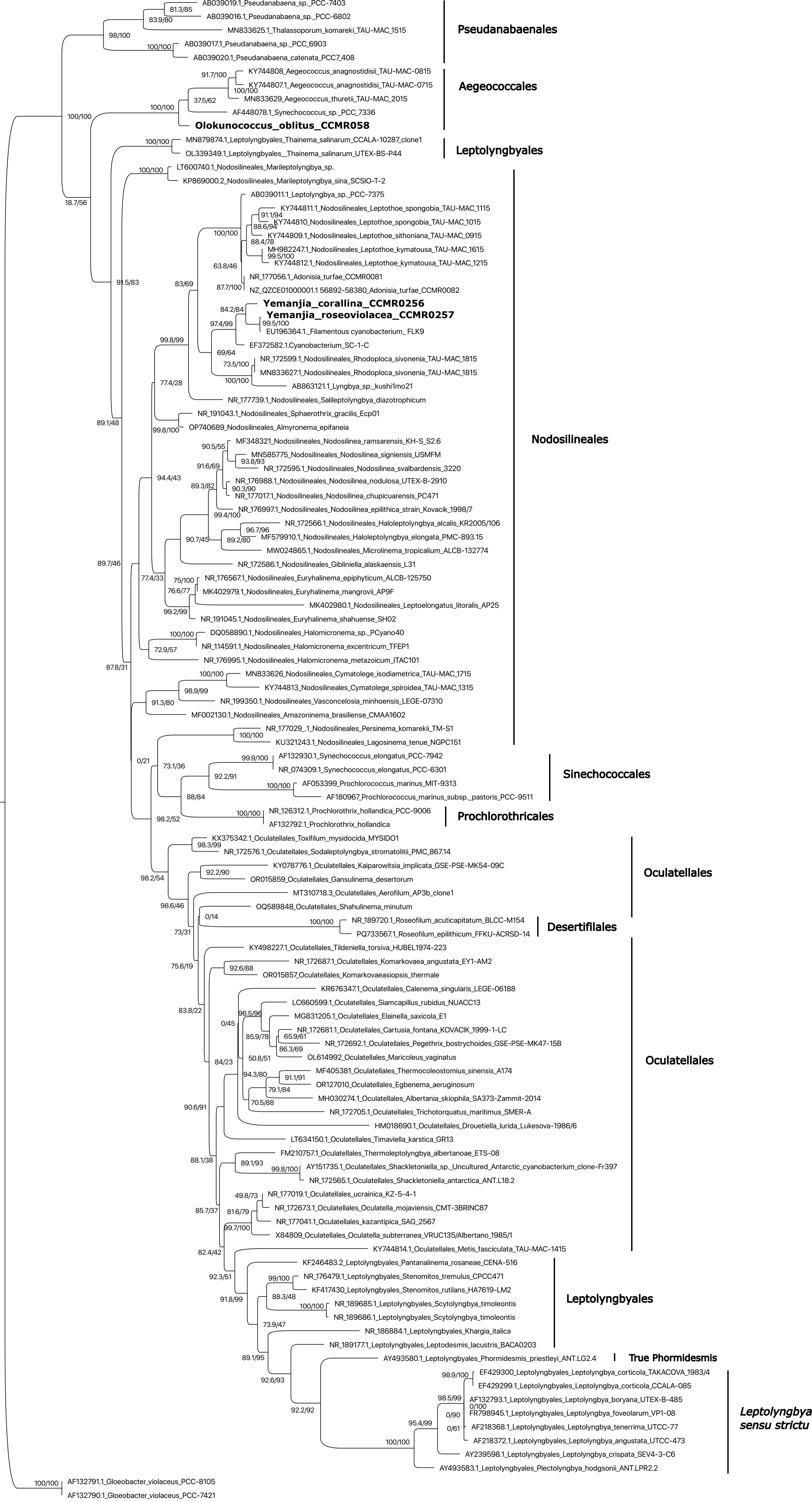

Supplement: Supplementary file 1 — Figure S1. Expanded 16S rRNA gene phylogenetic tree showing the position of the new taxa (names colored in bold) within the orders Nodosolineales and Aegeococcales. The tree was constructed with maximum likelihood method including sequences belonging to seven orders and Gloeobacter violaceus as outgroup. All available sequences of Nodosolineales and Aegeococcales were included as well as a more representative subset of Leptolyngbyales (relative to Figure 4) and Roseofilum sequences. Bootstrap support followed by SH‐like approximate likelihood ratio is shown at nodes. Accession numbers are shown in brackets. [file JPY-62-533-s006.pdf]

**a**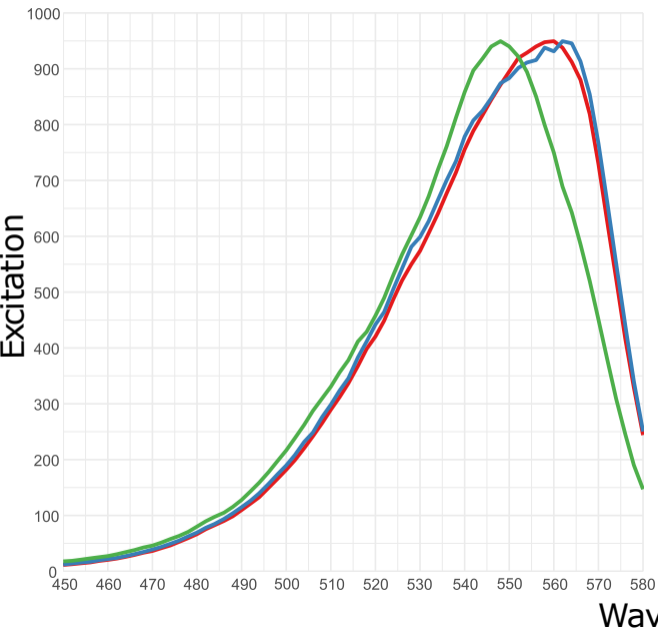**b**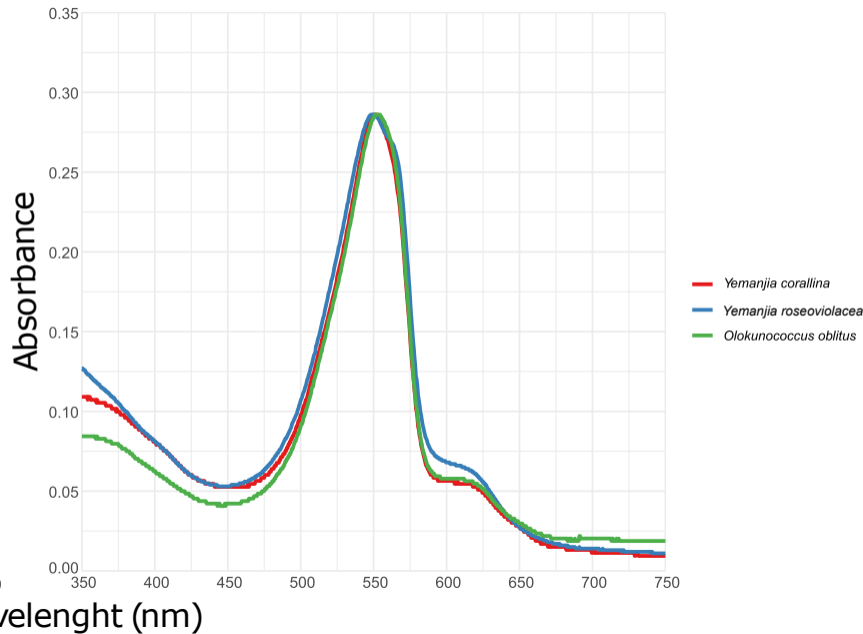

Supplement: Supplementary file 2 — Figure S2. Fluorescence‐excitation (a) and absorption (b) spectra of phycoerythrins from Yemanjia corallina, Y. roseoviolacea, and Olokunococcus oblitus. Spectra were normalized at the excitation maximum. A single well‐defined phycoerythrobilin peak was observed in all isolates, with no clear signal attributable to phycourobilin. [file JPY-62-533-s003.pdf]
