# Supplementary material for: Revealing novelty from the southwestern Atlantic, Yemanjia gen. nov. and Olokunococcus gen. nov. from the coral cyanobiome of the Abrolhos Bank
Source: J Phycol. 2026 Apr 23;62(2):533–55. doi: 10.1111/jpy.70159 (PMC13103685; doi:10.1111/jpy.70159)
Supplement: Supplementary file 3 — Table S1. Primers and amplification conditions [file JPY-62-533-s001.docx]

| **Gene** | **Primer** | **Species** | **Primer sequence** | **Annealing**  **temperature** | **References** |
| --- | --- | --- | --- | --- | --- |
| 16S | 106 F | *Olokunococcus oblitus*  *Yemanjia corallina*  *Y. roseoviolacea* | CGGACGGGTGAGTAACGCGTGA | 60 | Nübel *et al*., 1997 |
|  | 781R | *Olokunococcus oblitus* | GACTACWGGGGTATCTAATCCCAWTT | 60 | Nübel *et al*., 1997 |
|  | 359F | *Olokunococcus oblitus* | GGGGAATYTTCCGCAATGGG | 60 | Nübel *et al*., 1997 |
|  | 1093R | *Olokunococcus oblitus*  *Yemanjia corallina*  *Y. roseoviolacea* | GGGTTGCGCTCGTTGCGGGA | 60 | Turner *et al*., 1999 |
|  | 926 F | *Olokunococcus oblitus*  *Yemanjia corallina*  *Y. roseoviolacea* | AAACTCAAAGGAATTGACGG | 55 | De Gregoris *et al*., 2011 |
|  | ITER | *Olokunococcus oblitus*  *Yemanjia corallina*  *Y. roseoviolacea* | CTCTGTGTGCCTAGGTATCC | 55 | Wilmotte *et al*., 1993 |
| *rbc*L | rbcL3F | *Olokunococcus oblitus* | GACTTCACYAAAGACGACGAAA | 55 | Dvořák *et al*., 2014 |
|  | rbcL3R | *Olokunococcus oblitus* | CGRCCTTCGTTACGWGCTTG | 55 |  |
|  | *rbclf* | *Yemanjia corallina* | GACTTCACCAAAGAYGACGAAAACAT | 56 | Singh *et al*., 2015 |
|  | *rbclr* | *Yemanjia corallina* | GAACTCGAACTTRATYTCTTTCCA | 56 |  |
| *rpo*C1 | RF | *Yemanjia corallina* | TGGGGHGAAAGNACAYTNCCTAA | 60 | Seo and Yokota, 2003 |
|  | RR | *Yemanjia corallina* | GCAAANCGTCCNCCATCYAAYTGBA | 60 |  |
| ITS2 | 322 (F) | *Olokunococcus oblitus*  *Yemanjia corallina*  *Y. roseoviolacea* | TGTACACACCGCCCGTC | 55 | Iteman *et al*., 2000 |
|  | 340 (R) | *Olokunococcus oblitus*  *Yemanjia corallina*  *Y. roseoviolacea* | CTCTGTGTGCCTAGGTATCC | 55 |  |

**References**

De Gregoris, T. B., Aldred, N., Clare, A. S., & Burgess, J. G. (2011). Improvement of phylum- and class-specific primers for real-time PCR quantification of bacterial taxa. *Journal of Microbiological Methods, 86*(3), 351–356. https://doi.org/10.1016/j.mimet.2011.06.010

Dvořák, P., Casamatta, D. A., Poulíčková, A., Hašler, P., & Mareš, J. (2014). Morphological and molecular studies of *Neosynechococcus sphagnicola*, gen. et sp. nov. (Cyanobacteria, Synechococcales). *Phytotaxa, 170*(1), 3–38. https://doi.org/10.11646/phytotaxa.170.1.3

Iteman, I., Rippka, R., Tandeau de Marsac, N., & Herdman, M. (2000). Comparison of conserved structural and regulatory domains within divergent 16S–23S rRNA spacer sequences of cyanobacteria. *Microbiology, 146*(6), 1275–1286. https://doi.org/10.1099/00221287-146-6-1275

Nübel, U., Garcia-Pichel, F., & Muyzer, G. (1997). PCR primers to amplify 16S rRNA genes from cyanobacteria. *Applied and Environmental Microbiology, 63*(8), 3327–3332. https://doi.org/10.1128/aem.63.8.3327-3332.1997

Seo, P. S., & Yokota, A. (2003). The phylogenetic relationships of cyanobacteria inferred from 16S rRNA, *gyr*B, *rpo*C1 and *rpo*D1 gene sequences. *Journal of General and Applied Microbiology, 49*(4), 191–203. https://doi.org/10.2323/jgam.49.191

Singh, S. P., Rastogi, R. P., Häder, D.-P., & Sinha, R. P. (2015). Molecular characterization and phylogenetic analysis of cyanobacteria using rbcL gene sequences. *Annals of Microbiology, 65*(2), 799–807. https://doi.org/10.1007/s13213-014-0920-1

Turner, S., Pryer, K. M., Miao, V. P. W., & Palmer, J. D. (1999). Investigating deep phylogenetic relationships among cyanobacteria and plastids by small subunit rRNA sequence analysis. *Journal of Eukaryotic Microbiology, 46*(4), 327–338. https://doi.org/10.1111/j.1550-7408.1999.tb04612.x

Wilmotte, A., Van der Auwera, G., & De Wachter, R. (1993). Structure of the 16S ribosomal RNA of the thermophilic cyanobacterium *Chlorogloeopsis HTF* (‘m*astigocladus* *laminosus* HTF’) strain PCC 7518, and phylogenetic analysis. *FEBS Letters, 317*(1–2), 96–100. 10.1016/0014-5793(93)81499-P
