# Supplementary material for: Revealing novelty from the southwestern Atlantic, Yemanjia gen. nov. and Olokunococcus gen. nov. from the coral cyanobiome of the Abrolhos Bank
Source: J Phycol. 2026 Apr 23;62(2):533–55. doi: 10.1111/jpy.70159 (PMC13103685; doi:10.1111/jpy.70159)
Supplement: Supplementary file 4 — Table S2. rpoC1 region gene sequences identities (%) between Yemanjia corallina and its closest phylogenetic neighbors. Type strains are in bold. [file JPY-62-533-s002.pdf]

Yemanjia corallina and related sequences	1	2	3	4	5	6	7	8	9	10	11	12
**1. *Yemanjia corallina* CCMR0256**	100											
2. *Nodosilinea epilithica* LK55	80.18	100										
3. *Leptolyngbya* sp. PCC 9821	78.69	88.97	100									
4. *Nodosilinea nodulosa* PCC 7104	79.76	86.65	86.21	100								
5. *Leptolyngbya antarctica* ULC S47	78.54	87.08	86.5	86.21	100							
6. *Leptolyngbya fragilis* IFBC Pho03	79.15	82	82	81.57	83.31	100						
7. *Leptolyngbya* sp. PCC 7375	78.56	79.97	77.5	77.36	78.37	79.1	100					
8. *Leptolyngbya* sp. PCC 8104	78.23	79.97	77.5	77.36	78.37	79.1	100	100				
**9. *Leptothoe sithoniana* TAU-MAC 0915**	78.12	79.15	77.79	78.23	78.96	79.25	85.47	84.47	100			
**10. *Leptothoe kymatousa* TAU-MAC 1215**	76.95	78.33	76.78	78.08	77.79	77.94	80.83	80.26	82.62	100		
11. *Leptothoe kymatousa* TAU-MAC 1615	76.95	78.05	76.92	78.23	77.94	78.08	80.88	80.55	82.68	99.25	100	
**12. *Leptothoe spongobia* TAU-MAC 1015**	72.85	73.55	71.93	74.25	73.09	71.46	79.35	79.35	81.44	77.73	77.96	100
13. *Leptothoe spongobia* TAU-MAC 1115	71.84	70.26	69.38	72.42	70.25	70.1	75.29	75.18	77.59	72.84	72.99	82.83
