# Supplementary material for: Revealing novelty from the southwestern Atlantic, Yemanjia gen. nov. and Olokunococcus gen. nov. from the coral cyanobiome of the Abrolhos Bank
Source: J Phycol. 2026 Apr 23;62(2):533–55. doi: 10.1111/jpy.70159 (PMC13103685; doi:10.1111/jpy.70159)
Supplement: Supplementary file 5 — Table S3. rbcL gene sequences identities (%) between the closest phylogenetic neighbors of Yemanjia corallina and Olokunococcus oblitus (CCMR0258). Type strains are in bold. [file JPY-62-533-s004.pdf]

Yemanjia corallina and related sequences	1	2	3	4	5	6	7	8
**1. *Yemanjia corallina* CCMR256**	100							
**2. *Rhodoploca sivonenia* TAU-MAC 1815T**	86.53	100						
**3. *Cymatolege isodiametrica* TAU-MAC 1715**	85.16	88.19	100					
**4. *Leptothoe kymatousa* TAU-MAC 1215**	83.14	89.85	85.98	100				
**5. *Cymatolege spiroidea* TAU-MAC 1315**	83.88	88.75	92.83	88.13	100			
**6. *Leptothoe sithoniana* TAU-MAC 0915**	85.31	86.9	85.74	86.14	84.04	100		
7. *Halomicronema excentricum* Lakshadweep	84.38	86.53	85.14	84.31	85.99	84.55	100	
**8. *Leptothoe spongobia* TAU-MAC 1015**	82.19	87.27	83.95	90.48	84.2	89	85.14	100
9. *Nodosilinea epilithica* ACSSI 169	83.75	84.5	84.25	84.97	84.85	83.36	88.47	84.25
***Olokunococcus oblitus* and related sequences**	**1**	**2**	**3**	**4**	**5**	**6**	**7**	**8**
**1. *Olokunococcus oblitus* CCMR258**	100							
**2. *Aegeococcus anagnostidisi* TAU-MAC 0815**	90.79	100						
**3. *Aegeococcus thureti* TAU-MAC 2015**	90.54	95.04	100					
4. *Pseudanabaena* sp. PCC 6802	83.21	84.4	83.1	100				
**5. *Leptolyngbya laminosa* ETS-08**	83.21	81.58	83.1	82.76	100			
6. *Pseudanabaena* sp. PCC 7367	81.13	83.21	82.64	85.31	79.79	100		
**7. *Synechococcus elongatus* PCC 6301**	81.58	82.17	81.71	80.24	81.87	78.9	100	
8. *Neosynechococcus sphagnicola* sy1	80.39	80.39	81.55	81.72	83.8	80.83	80.98	100
**9. *Thalassoporum komareki* TAU-MAC 1515**	78.95	81.5	81.09	84.21	78.65	92.93	78.5	79.55
