# Supplementary material for: Revealing novelty from the southwestern Atlantic, Yemanjia gen. nov. and Olokunococcus gen. nov. from the coral cyanobiome of the Abrolhos Bank
Source: J Phycol. 2026 Apr 23;62(2):533–55. doi: 10.1111/jpy.70159 (PMC13103685; doi:10.1111/jpy.70159)
Supplement: Supplementary file 6 — Table S4. Annotation of the 16S–23S ITS rRNA regions and corresponding lengths. [file JPY-62-533-s005.docx]

| **Strain** | **Leader** | **D1-D1’ Helix** | **Spacer with D2** | **Spacer and D3 with Spacer** | **tRNA-Ile** | **Spacer with V2 Helix and spacer** | **tRNA-Ala** | **Spacer** |
| --- | --- | --- | --- | --- | --- | --- | --- | --- |
| *Yemanjia corallina* | 6 | 62 | 59 | 23 | 74 | 6 | 76 | 34 |
| *Yemanjia roseoviolacea* | 6 | 62 | 29 | 23 | 74 | 6 | 76 | 65 |
| *Olokunococcusoblitus* | 6 | 79 | 71 | 32 | 74 | 46 | 76 | 33 |

| **Strain** | **Box B** | **Spacer to end of Box A** | **D4** | **Spacer** | **V3 + end region** | **Total length** |
| --- | --- | --- | --- | --- | --- | --- |
| *Yemanjia corallina* | 27 | 29 | 7 | 24 | 67 | **494** |
| *Yemanjia roseoviolacea* | 36 | 34 | 7 | 26 | 71 | **515** |
| *Olokunococcus oblitus* | 36 | 30 | 7 | 7 | 126 | **623** |
